# Recurrence of mogamulizumab-associated rash in patients with relapsed erythrodermic cutaneous T-cell lymphoma after retreatment with mogamulizumab

**DOI:** 10.1016/j.jdcr.2025.06.049

**Published:** 2025-07-25

**Authors:** Emma R. McIntyre, Liliana Crisan, Jasmine Zain, Christiane Querfeld

**Affiliations:** a Division of Dermatology, City of Hope National Medical Center, Duarte, California; b College of Medicine, California Northstate University, Elk Grove, California; c Beckman Research Institute, City of Hope National Medical Center, Duarte, California; d Department of Hematology and Hematopoietic Cell Transplantation, City of Hope National Medical Center, Duarte, California; e Department of Pathology, City of Hope National Medical Center, Duarte, California

**Keywords:** cutaneous T-cell lymphoma, mogamulizumab, mogamulizumab-associated rash, mycosis fungoides, Sézary syndrome

## Introduction

Mycosis fungoides (MF), and the leukemic variant Sézary syndrome (SS) are the most common types of cutaneous T-cell lymphomas (CTCL) characterized by a chronic course with limited therapeutic options for advanced stages of disease.^1^ Mogamulizumab (moga) is a monoclonal antibody therapy with enhanced antibody-dependent cellular cytotoxicity activity against the C-C motif chemokine receptor 4 receptor that is expressed on malignant T cells, facilitating their migration to the skin.^2^ Moga was Food and Drug Administration approved in 2018 for relapsed/refractory MF and SS.^3^ In addition to its direct cytotoxic effects against malignant T cells, moga has immunomodulatory capabilities by decreasing the number of immunosuppressive T regulatory cells.^4^ Mogamulizumab-associated rash (MAR) is one of the most common adverse effects of moga, with 24% of patients with MF/SS in the phase 3 moga trial experiencing drug eruption.^5^ Several studies have shown that MAR incidence is higher in treatment responders.^6^^,^^7^ Trum et al^7^ also found that both MAR and clinical response to moga may be more likely in patients with higher blood disease burden (ie, SS vs MF). The mechanism of MAR, and its higher frequency in SS and treatment responders is unclear but thought to be due to unleashed immune response because of T regulatory cell depletion.^7^ MAR is graded by Common Terminology Criteria for Adverse Events for skin and subcutaneous tissue disorders.^8^ Recommended therapies for MAR include topical corticosteroids for rashes grades 1, topical steroids and consideration of tapered oral steroids, low-dose methotrexate or dupilumab for rashes grade 2, and for grade 3 rash, moga should be held, and a topical and systemic corticosteroid taper should be started right away and then consider the same systemic therapies.^6^^,^^7^ Although MAR is typically reversible and mild to moderate in severity, there is little information regarding the nature of recurrent MAR after retreatment with moga, as well as efficacy of retreatment with moga.^9^ The purpose of this study was to evaluate and describe the experience and impact of retreatment of moga on previously treated patients who experienced MAR.

## Methods

We conducted a retrospective chart review of patients with erythrodermic CTCL who were treated with moga from 2019 to 2024 at a single comprehensive cancer referral center. In total, 48 patients were treated with standard of care mogamulizumab; 25 of them experienced MAR during the first moga treatment; 4 of 25 patients were retreated with moga because of disease recurrence in skin and blood, and all 4 patients experienced recurrent MAR. For these 4 patients, data were collected to include patient demographics as well as the timing and duration of treatment, response, and onset, duration and severity of MAR.

## Results

**Fig 1:**
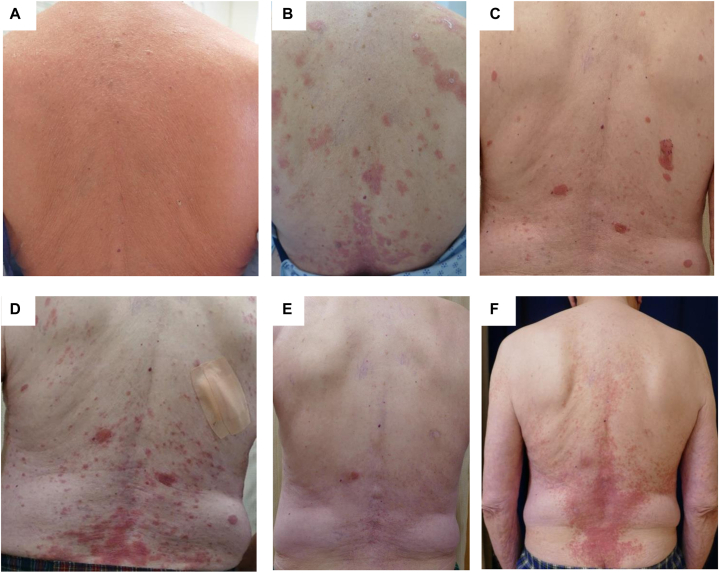
Clinical images of patient 1 with Sézary syndrome. Clinical image at baseline before mogamulizumab treatment **(A),** the occurrence of mogamulizumab-associated rash (MAR) presenting with psoriasiform plaques during first moga treatment course **(B),** relapsed disease presenting with erythematous plaques **(C),** MAR with subsequent moga retreatment **(D),** achievement of complete remission **(E),** and followed by new MAR onset **(F)**.

Herein we report 4 patients with erythrodermic CTCL (SS, *n* = 3; erythrodermic MF, *n* = 1), who all experienced MAR and achieved complete remission after their first moga treatment and were retreated with moga following disease relapse, with variable development of MAR and remission status after retreatment. All patients had advanced stage MF or SS (Fig 1, *A*) and had previously tried at least 1 systemic treatment before beginning moga treatments (Table I). Time to onset of MAR ranged between 2 and 5 months. The average duration of the first moga treatment was 3 months (SD: 1.2), around which time all patients experienced their first MAR (Fig 1, *B*). The first occurrence of MAR lasted on average 7 months (SD: 2.9) and was grade 2 for all patients.^8^ The first MAR resolved with low-dose (15 mg) weekly oral methotrexate and topical corticosteroids in patient 1 and 2, topical corticosteroids in patient 3, and oral plus topical corticosteroids and low-dose (15 mg) oral methotrexate in patient 4. All patients achieved complete CTCL remission with a mean time of 6.5 months after their first dose of moga (SD: 2.5). Patients maintained complete remission after the first moga treatment course with a mean time of 13 months (SD: 1.1).

**Table I:** Patient demographics, mogamulizumab treatment courses, and responses and outcomes

Demographic, clinical and treatment characteristics		Patient 1	Patient 2	Patient 3	Patient 4
Baseline	Sex	Male	Male	Female	Female
	Diagnosis	SS	SS	Erythrodermic MF	SS + LCT
	TNMB stage	IVA1, T4N0M0B2b	IVA2, T4N3M0B2b	IIIB, T4N0M0B1b	IVA1 T4N0MB2b
	Age at diagnosis (y)	85	79	80	50
	mSWAT score	100	80	80	50
	Topical treatments (*n*)	1	2	1	1
	Systemic treatments (*n*)	1	1	2	1
First moga tx	Treatment duration (mo)	2	3	5	2
	(*n*) Cycles, (*n*) infusions	3, 7	4, 10	6, 10	3, 7
	TTO of MAR (mo)	2	4	5	2
	MAR grade	2	2	2	2
	MAR duration (mo)	2	9	9	8
	Best response	CR	CR	CR	CR
	TTO of CR (mo)	3	6	7	10
	CR duration (mo)	15	13	13	12
Second moga tx	Treatment duration (mo)	7	4	3	3
	Additional systemic treatment	Methotrexate	Methotrexate	N/A	N/A
	(*n*) Cycles, (*n*) infusions	7, 15	5, 10	3, 8	3, 8
	TTO of MAR (mo)	9	5	3	8
	Duration of MAR (mo)	4	3	10	3
	MAR grade	2	1	1	2
	Best response	CR	CR	PR	CR
	TTO of CR	10	10	N/A	2
	Duration of CR/PR (mo)	12+	13+	20	3+
Third moga tx	Treatment duration (mo)	N/A	N/A	1	N/A
	(*n*) Cycles, (*n*) infusions	N/A	N/A	3, 3	N/A
	Best response	N/A	N/A	PD	N/A

*CR*, Complete remission; *LCT*, large cell transformation; *MAR*, mogamulizumab-associated rash; *MF*, mycosis fungoides; *mSWAT*, modified Severity-Weighted Assessment Tool; *N/A*, not applicable; *PD*, progressive disease; *PR*, partial remission; *SS*, Sézary syndrome; *TNMB*, tumor, lymph node, metastasis, blood; *TTO*, time to onset; *tx*, treatment.

Upon CTCL relapse (Fig 1, *C*), patient 1 and 2 were treated with oral methotrexate alone, then methotrexate plus second treatment moga once their disease worsened. Patient 3 and 4 started the second treatment of moga without additional systemic treatment after CTCL relapse. Time to onset of MAR ranged between 1 and 2 months. On average, second treatment with moga lasted 4 months (SD: 1.6), with MAR following up to 8 months after the last treatment in all patients (Fig 1, *D*). Mean time of duration of second MAR in all patients lasted 5 months (SD: 3), and was grade 2 in patient 1 and 4, and grade 1 in patients 2 and 3. The second MAR resolved with topical and oral corticosteroids in patient 1 (Fig 1, *E*), corticosteroids and methotrexate in patient 2, dupilumab, triamcinolone ointment, and methotrexate in patient 3, and corticosteroids in patient 4. Of note, patient 1 experienced 2 additional episodes of MAR after his second treatment course with moga, which resolved with low-dose methotrexate and corticosteroids (Fig 1, *F*). Patients 1, 2, and 4 were able to achieve complete CTCL remission on average 7 months after starting the second treatment with moga (SD: 3.8). However, patient 3 was only able to obtain partial remission. On average, the duration of the best response to the second moga treatment (either partial or complete remission) was 12 months (SD: 6). In general, oral methotrexate for MAR was given at low dose at 15 mg weekly.

Twenty months after the second moga treatment, patient 3 experienced worsening of CTCL and underwent a third treatment with moga. One month into the third treatment with moga, she experienced numerous erythematous patches and thin plaques, histologically consistent with CTCL, prompting moga discontinuation. Her disease progressed to SS stage IVA1 (T4N0M0B1b) despite trials of other therapeutic options.

## Discussion

Retreatment with moga in patients with relapsed CTCL has been sparsely reported with varying levels of success.^10^ Furthermore, there is a dearth of literature regarding MAR after moga rechallenge. We found that retreatment with moga is safe and efficacious, as none of our patients experienced serious adverse events, and 3 out of 4 patients reobtained complete remission after the second treatment with moga. Treatment-emergent C-C motif chemokine receptor 4 mutations or loss of C-C motif chemokine receptor 4 expression have been associated with disease resistance to moga,^11^ and thus it is possible that patient 3 experienced disease progression due to the potential resistance mechanisms after his third treatment course.

MAR developed in all 4 patients after the first and second moga treatment course. One patient did not experience MAR after the third retreatment with moga and showed lack of response during this treatment course, which is consistent with previous studies that found MAR is much more likely to occur in treatment responders.^5^, ^6^, ^7^ A recent study also investigated the occurrence of MAR in patients retreated with moga combined with concurrent or sequential methotrexate, acitretin, or total electron beam therapy.^12^ Similar to our results MAR reoccurred only in responding patients, but not in all retreated responding patients, which may be due to concomitant or sequential combination regimen. The lower incidence of MAR in these patients could be due to additional effects on the CTCL microenvironment; particularly, radiation or methotrexate can affect T regulatory cell function and macrophages, making them less likely to develop an unleashed reaction.

Time to onset of MAR after moga initiation was highly variable in all our patients and occurred within 2 to 7 months of treatment but appeared to reoccur earlier during retreatment courses. MAR can recur with every moga retreatment and may resolve with temporary moga cessation, topical or oral corticosteroids, and oral low-dose weekly methotrexate. In our experience weekly doses of 15 mg orally were efficient to treat MAR.

Methotrexate plays an important role in treating both MAR and CTCL. Methotrexate is an antimetabolite, thereby slowing the progression of CTCL, but it also has anti-inflammatory properties due to its ability to increase T regulatory suppressive function and suppress autoimmunity.^13^ The immune-modulating effects of methotrexate were observed in patient #1, who experienced MAR within 2 months of starting the first moga treatment but was MAR-free for 9 months after starting the second moga treatment with methotrexate.

In conclusion, our small case series provides evidence that retreatment with moga is generally well tolerated, with patients responding positively to rechallenge. MAR can recur with every retreatment and appears to correlate with response and is similar in severity during subsequent courses. MAR may be mitigated with concomitant low-dose weekly methotrexate and can be continued on moga treatment.

## Conflicts of interest

Dr Querfeld has served on advisory board/steering committees for Kyowa Kirin, 10.13039/100008130Helsinn Therapeutics (US) Inc, Citius Pharmaceuticals, and SLAM BioTherapeutics and has received research grants from Kyowa Kirin and Helsinn. The other authors have no conflicts of interest to declare.
